# Microbiome structure of the fungid coral *Ctenactis echinata* aligns with environmental differences

**DOI:** 10.1111/mec.13251

**Published:** 2015-06-19

**Authors:** Cornelia Roder, Till Bayer, Manuel Aranda, Maren Kruse, Christian R. Voolstra

**Affiliations:** ^1^ Red Sea Research CenterKing Abdullah University of Science and TechnologyThuwal23955‐6900Saudi Arabia; ^2^ GEOMAR Helmholtz Centre for Ocean ResearchKiel24105Germany; ^3^ Leibniz Center for Tropical Marine EcologyBremen28359Germany

**Keywords:** coral reef, ecological niche, holobiont, metaorganism, microbiome, symbiosis

## Abstract

The significance of bacteria for eukaryotic functioning is increasingly recognized. Coral reef ecosystems critically rely on the relationship between coral hosts and their intracellular photosynthetic dinoflagellates, but the role of the associated bacteria remains largely theoretical. Here, we set out to relate coral‐associated bacterial communities of the fungid host species *Ctenactis echinata* to environmental settings (geographic location, substrate cover, summer/winter, nutrient and suspended matter concentrations) and coral host abundance. We show that bacterial diversity of *C. echinata* aligns with ecological differences between sites and that coral colonies sampled at the species’ preferred habitats are primarily structured by one bacterial taxon (genus *Endozoicomonas*) representing more than 60% of all bacteria. In contrast, host microbiomes from lower populated coral habitats are less structured and more diverse. Our study demonstrates that the content and structure of the coral microbiome aligns with environmental differences and denotes habitat adequacy. Availability of a range of coral host habitats might be important for the conservation of distinct microbiome structures and diversity.

## Introduction

Recent advancements in sequencing technology have led to a new understanding of the role of micro‐organisms in shaping animal biology emphasizing the diversity and functional capacity of bacteria, and challenging our views on what constitutes a genome or an organism (McFall‐Ngai *et al*. 2013). While tropical shallow water corals have long been recognized to exist in close and obligate relationships with endosymbiotic unicellular algae (also referred to as zooxanthellae) of the genus *Symbiodinium* (Muscatine & Cernichiari 1969), the importance of the diverse community of bacteria became only recently established (Rosenberg *et al*. 2007). This functional metaorganism consisting of the coral animal host, its photosynthetic algal symbionts, and microbial assemblage is termed the coral holobiont (Rosenberg *et al*. 2007). Coral‐associated bacteria are shown to confer immunity (Kelman *et al*. 2006) and to support the host's metabolic demands (Lesser *et al*. 2004). They are rich in abundance as well as in diversity (Rohwer *et al*. 2002) and differ between holobiont compartments such as tissue, mucus or skeleton (Li *et al*. 2014). Their diversity furthermore differs from that of assemblages present in the surrounding water column (Frias‐Lopez *et al*. 2002; Roder *et al*. 2014) and the prevailing bacterial community is host species specific (Rohwer *et al*. 2002; Sunagawa *et al*. 2010). And even though intracolonial variation has been documented (Li *et al*. 2013), bacterial assemblages associated with corals are well structured with distinct operational taxonomic units (OTU) frequently being highly abundant (Rohwer *et al*. 2001; Bayer *et al*. 2013a,b). Differences in microbial communities across coral species are assumed to be due to different corals associating with different microbes of similar function rather than phylogenetic affiliation (Li *et al*. 2013; Kelly *et al*. 2014). Nevertheless, changes in response to season (Littman *et al*. 2009) or geographic location (Koren & Rosenberg 2006) have been documented, and coral‐associated bacterial communities are sensitive towards environmental insult, experiencing large shifts during bleaching or disease (Bourne *et al*. 2008; Roder *et al*. 2014). It has been proposed that dynamics in coral‐associated microbial populations are an important mechanism for the holobiont to rapidly acclimate to changes in the environment (Reshef *et al*. 2006), but to which degree a coral's microbiome is structured by environmental conditions, temporal factors, or host phylogeny and physiology remains elusive and, to date, a comprehensive approach analysing coral microbiota dynamics in space and time is lacking.

Here, we set out to explore the variability of bacteria associated with the fungid coral *Ctenactis echinata* during summer and winter and across four habitats in the central Red Sea to further understand how environmental conditions and the coral microbiome structure relate. To do this, we ecologically described fore‐ and back‐reef environments of nearshore and offshore coral reefs detailing substrate condition, water temperature, nutrients, suspended matter concentrations and abundance of *C. echinata* and we compared these data to the associated bacterial communities of *C. echinata* across all sites.

## Materials and methods

### Sampling

**Figure 1:**
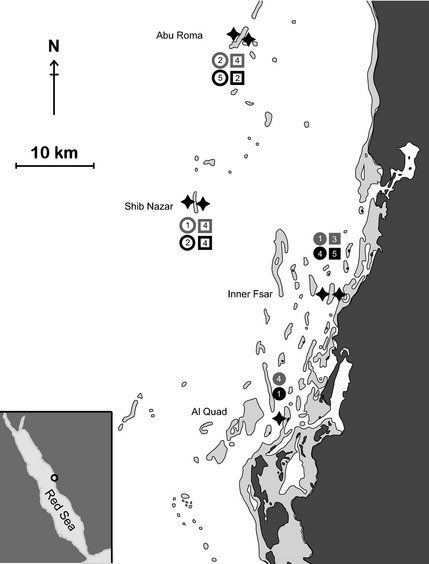
Map of study sites. Offshore (Abu Roma and Shib Nazar) and nearshore (Al Quad and Inner Fsar) coral reef sites were sampled from exposed (i.e. ocean facing) and sheltered (i.e. land facing) habitats (indicated by stars) on two sampling occasions (i.e. summer and winter). Replicate numbers are shown for each sampling event and site. Open symbols: offshore, closed symbols: nearshore, circles: exposed reef sites, squares: sheltered reef sites; grey: summer, black: winter.

Between 1 and 5 whole unattached and visually healthy polyps of *Ctenactis echinata* of equivalent size classes (<10 cm length) were collected across four reefs and their respective fore‐ and back‐reef environments over two sampling dates at 4–7 m depth using SCUBA in the central Red Sea. Each of two reefs denoted nearshore (i.e. Inner Fsar and Al Quad) and offshore (i.e. Abu Roma and Shib Nazaar) environments and were combined to provide between three and eight coral samples for any combination of fore‐reef or back‐reef and nearshore or offshore environments (Fig. 1). Details on sampling location, transect and environmental data collection and bacterial community sampling are provided as supplementary information (Table S1, Supporting information). Sampling took place on two occasions, once during summer (August 2011) and once during winter (February 2012) along exposed fore‐reef and sheltered back‐reef sides of two offshore (>25 km distance to shore) and two nearshore (<5 km distance to shore) reefs (Table S1, Supporting information). Samples were handled by wearing gloves and immediately transferred into sterile Whirl‐Pak bags after collection. Upon arrival on board, samples were rinsed with filtered (0.22 μm) sea water to remove loosely associated microbes. Samples were subsequently wrapped in aluminium foil and flash frozen in liquid nitrogen until analysis. At each sampling site, a water sample was collected in sterile cubitainers (1 L) and kept on ice until further processing (see below). Temperature at the study sites during sampling was recorded using a conventional thermometer.

### Transect data

Reef substrate was characterized according to live (‘hermatypic corals’ and ‘other live cover’, that is soft corals, macro/micro/turf/calcifying algae, sponges and anemones) and dead (‘bare firm substrate’ and ‘loose substrate’) cover using the line intercept method (Hill & Wilkinson 2004) in 0.5 m distances along four 20‐m segments (separated by 5 m intervals) of a 100‐m transect at each sampling site. At one of the nearshore and one of the offshore sites, the abundance of *C. echinata* was counted in 2‐m‐wide belts (Hill & Wilkinson 2004) along four 20‐m transects at both sides of the reefs, that is the exposed (fore‐reef) and sheltered (back‐reef) side (Table S1, Supporting information).

### Sample processing

Water samples were filtered onto 0.22‐μm Isopore filters (Millipore) for gravitational determination (Mettler Toledo XS205) of total suspended matter (TSM) and for DNA extraction of the associated microbial community. *C. echinata* tissue was removed from the coral skeleton using pressurized air. DNA from water and coral samples was extracted according to the manufacturer's instructions using the QIAGEN DNeasy Plant Mini Kit. Variable regions 5 and 6 of the 16S rRNA gene were amplified using the 784F and 1061R primer pair (Andersson *et al*. 2008) containing barcodes and Roche 454 pyrosequencing adaptors for subsequent library construction as detailed in Bayer *et al*. (2013b) and Hamady *et al*. (2008). Polymerase chain reaction (PCR) was prepared using the QIAGEN Multiplex PCR kit with 0.2 μm of each primer and 2 ng DNA for water samples or 30 ng DNA for coral samples plus DNA/RNA‐free water (TEKnova) to a final PCR volume of 25 μL. Temperature cycling profile for amplification was as follows: 95 °C for 15 min followed by 27 cycles of 95 °C for 30 s, 55 °C for 40 s and 72 °C for 40 s, followed by one cycle at 72 °C for 10 min. For each sample, amplifications were performed in triplicate and combined. PCR products for all samples were quantified using a microplate reader (SpectraMax Paradigm; Molecular Devices) and the Qubit Broad Range assay (Invitrogen) prior to pooling of all samples in equal quantities. Sequencing was performed on the Roche 454 FLX platform. Inorganic nutrient concentrations (nitrate + nitrite, nitrite, ammonia, phosphate and silicate) in the filtrate of the water samples were determined using standard colorimetric tests and a QuickChem 8000 (Zellweger Analysis, Inc.) AutoAnalyzer.

### Data analysis

Sequencing data were analysed using the open‐source software mothur (Schloss *et al*. 2009). Sequence reads were split according to barcodes and quality trimmed prior to alignment against the silva database (silva ssu Release 102). Chimeric sequences were removed using the uchime program as implemented in mothur (Edgar *et al*. 2011) followed by preclustering of the data to 1‐bp difference to compact data and reduce OTUs generated by sequencing errors (Huse *et al*. 2010). Remaining singletons (i.e. sequences that were only present once across all samples) were removed from the data set yielding samples with a maximum of 12 503 sequence reads (median: 2013, mean 2939 reads per sample). To obtain a minimum of three colony replicates over the categories reef location, sheltered/exposed environment and summer/winter, data were subsampled to 500 sequence reads to allow for the inclusion of samples with low numbers of sequence reads. Detailed information on sequence counts, taxonomic classification and 16S reference amplicon sequences for all OTUs across all samples used in this study is available as supplemental data (Table S2, Supporting information). Sequence raw data determined in this study have been deposited in the NCBI Sequence Read Archive under accession no. PRJNA277291.

Bacterial assemblages associated with reef water and coral specimens were tested for differences between shelf sites (‘offshore’ vs. ‘nearshore’), exposures (‘exposed’ vs. ‘sheltered’) and time of year (‘summer’ vs. ‘winter’, referenced as ‘season’ in the following) using permutation multivariate analysis of variance (permanova). Here, all fixed factors (‘site’, ‘exposure’ and ‘season’) were nested according to hierarchy, and 999 permutations of residuals were conducted based on Bray–Curtis distances between samples using the primer‐e software with the permanova+ add‐on package (Clarke & Gorley 2006). Environmental differences in water quality (TSM, nutrients, temperature) between shelf sites (‘offshore’ vs. ‘nearshore’), exposures (‘exposed’ vs. ‘sheltered’) and time of year (‘summer’ vs. ‘winter’) were also identified applying permanova as above, but on Euclidean distances between samples. Here, primer‐e's similarity percentage analysis (simper) on Euclidean distances further revealed the main contributors of the parameters under investigation responsible for site and sampling date differences. Substrate cover data between sites was compared applying the anosim treatment based on Euclidean distances in primer‐e (Clarke & Gorley 2006).

## Results

### Environmental settings

Water quality (Table 1) between fore‐ and back‐reef environments of near‐ and offshore coral reefs (Fig. 1) differed significantly between summer and winter (*P *=* *0.002) and with distance from shore (*P *=* *0.02), but was similar for exposed and sheltered sides within the same reef locations (i.e. nearshore vs. offshore) (Table 2). Sampling date (i.e. summer vs. winter) differences were mainly driven by temperature, while nearshore and offshore reefs differed in concentration of TSM, and to a lesser extent in temperature (Table 2). Nutrient concentrations did not differ significantly between seasons or sites.

**Table 1:** Temperature and concentration of suspended matter and inorganic nutrients (nitrite + nitrate, nitrite, ammonia, phosphate and silicate) at sampling sites of the coral *Ctenactis echinata*

Reef name	Shelf	Exposure	Sampling date	Temp (°C)	Total suspended matter (TSM) (mg/L)	Nitrite + nitrate (μm)	Ammonia (μm)	Phosphate (μm)	Silicate (μm)	Nitrite (μm)
Inner Fsar	NS	Sheltered	Summer	33	4.27	0.50	0.65	0.08	0.56	0.07
Inner Fsar	NS	Sheltered	Winter	25	6.20	0.38	0.34	0.10	0.55	0.05
Inner Fsar	NS	Exposed	Summer	33	3.83	0.07	0.35	0.03	0.40	0.04
Inner Fsar	NS	Exposed	Winter	25	4.78	0.24	0.39	0.13	0.43	0.05
Al Quad	NS	Exposed	Summer	32	3.00	0.09	1.17	0.04	0.27	0.06
Al Quad	NS	Exposed	Winter	25	4.60	0.16	0.77	0.02	0.20	0.26
Abu Roma	OS	Sheltered	Summer	30	1.20	0.19	0.18	0.09	0.35	0.09
Abu Roma	OS	Sheltered	Winter	26	1.67	0.34	0.21	0.12	0.54	0.06
Shib Nazar	OS	Sheltered	Summer	31	3.07	0.45	0.42	0.08	0.71	0.04
Shib Nazar	OS	Sheltered	Winter	25	4.09	0.32	0.21	0.14	0.54	0.06
Abu Roma	OS	Exposed	Summer	30	1.11	0.84	0.15	0.10	0.49	0.06
Abu Roma	OS	Exposed	Winter	26	2.20	0.15	0.19	0.11	0.45	0.01
Shib Nazar	OS	Exposed	Summer	31	3.07	0.18	0.19	0.07	0.68	0.04
Shib Nazar	OS	Exposed	Winter	25	4.50	0.13	0.64	0.03	0.24	0.22

NS, nearshore; OS, offshore.

**Table 2:** Differences in environmental conditions between habitats and sampling dates of the coral *Ctenactis echinata*

permanova	d.f.	SS	MS	Pseudo‐F	Unique permutations	Monte Carlo *P*‐value
Shelf	1	15.96	15.96	7.31	998	0.020
Exposure (shelf)	2	2.29	1.14	0.52	999	0.691
Season [exposure(shelf)]	4	144.27	36.07	16.53	999	0.002
Residuals	6	13.09	2.18			
Total	13	174				

SIMPER						
Summer vs. winter average squared distance = 41.06						
		Summer average	Winter average	Av.Sq. Dist.	Sq.Dist/SD	Contrib %
Temperature (°C)		31.40	25.30	37.60	2.09	91.48

Nearshore vs. offshore average squared distance = 8.21						
		Nearshore average	Offshore average	Av.Sq. Dist.	Sq.Dist/SD	Contrib %
TSM (mg/L)		4.45	2.61	5.00	0.93	60.87
Temperature (°C)		28.80	28.00	2.83	0.89	34.51

Results of the permanova analysis showing differences between ‘shelf’ (i.e. reef locations: nearshore vs. offshore), ‘season’ (i.e. sampling date: summer vs. winter) and ‘exposure’ (i.e. fore‐/back‐reef environment: exposed vs. sheltered). Results of SIMPER analyses showing main factors contributing to a total of >90% of the observed differences between sampling dates and shelf locations, respectively.

**Figure 2:**
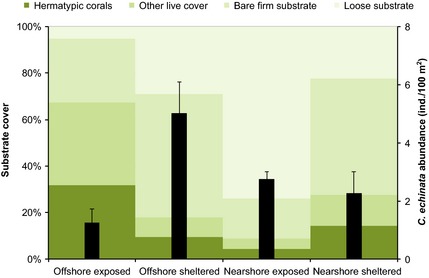
Substrate cover composition (coloured bars) and abundance (black bars) of the coral *Ctenactis echinata* at different habitats. Offshore sheltered habitats are the species’ preferred habitat and represent distinct environmental conditions. Error bars indicate SE.

Benthic cover composition differed significantly between all locations except between the sheltered sides of nearshore and offshore reefs (all sites *P*_ANOSIM_ = 0.001, Table S3, Supporting information). Live benthic cover was substantially higher in the exposed offshore reefs compared to all other habitats (Fig. 2). While dead substrate at nearshore and offshore sheltered sites mainly consisted of firm rock, the exposed sides of the nearshore reefs were mainly covered by loose substrate. Importantly, *Ctenactis echinata* had a distinct distribution pattern and was most abundant on the rocky sheltered sides of the offshore reefs and less present at the exposed sides of the same reefs or at nearshore reef sites (*P*_Kruskal–Wallis_ = 0.0371, Fig. 2).

### Temporal and spatial patterns of microbial communities

**Figure 3:**
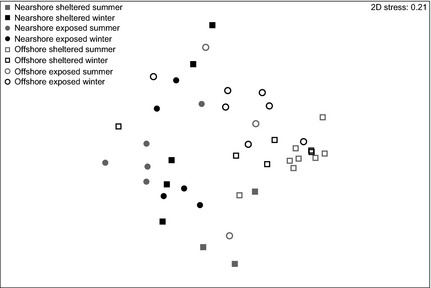
Nonmetric multidimensional scaling plot of bacterial communities associated with *Ctenactis echinata* samples. Bray–Curtis distances between samples illustrate differences between nearshore and offshore sites as well as between offshore sheltered and offshore exposed environments. Open symbols: offshore, closed symbols: nearshore, circles: exposed reef sites, squares: sheltered reef sites, grey: summer, black: winter.

To understand microbial assemblage patterns associated with *C. echinata* over different habitats and sampling dates, we analysed bacterial communities of coral colonies and the surrounding water column via 16S rRNA gene sequencing resulting in a total number of 850 distinct bacterial OTUs at the 0.03 level. Bacterial community profiles of reef water and coral tissue were highly different: only 90 bacterial taxa were encountered in both coral colonies and the water column, while 703 and 237 OTUs were solely associated with coral or water, respectively (Table 3). Further, bacterial communities of water samples showed a highly even distribution independent of sites and conditions (Pielou's evenness *J* mean = 0.76), whereas evenness of bacterial assemblages associated with corals differed between sites from 0.31 to 0.66 (Pielou's evenness *J* mean = 0.52) (Table 3). Bacterial assemblages associated with the reef water did not vary between sites, but between summer and winter (Table 4). In contrast, the bacterial diversity of *C. echinata* (Fig. 3) was highly different between sites as well as between the two sampling dates (i.e. summer vs. winter) (Table 4).

**Table 3:** Bacterial profiling of coral and water samples

Coral/Water	No. of samples	Total no. of OTUs	Average no. of OTUs	Pielou's evenness *J*	Shannon diversity *H*′
Nearshore					
Sheltered					
Summer	3	165	55 (±15)	0.60 (±0.20)	2.44 (±2.39)
Winter	5	304	61 (±22)	0.61 (±0.10)	2.44 (±2.82)
Exposed					
Summer	5	318	64 (±12)	0.61 (±0.14)	2.51 (±2.20)
Winter	5	312	62 (±21)	0.58 (±0.12)	2.36 (±2.86)
Offshore					
Sheltered					
Summer	8	179	22 (±4)	0.31 (±0.19)	0.99 (±1.71)
Winter	6	132	22 (±3)	0.31 (±0.19)	0.97 (±1.22)
Exposed					
Summer	3	151	50 (±15)	0.66 (±0.11)	2.58 (±2.40)
Winter	7	189	27 (±2)	0.49 (±0.21)	1.62 (±1.23)
Nearshore					
Sheltered					
Summer	1	75	75	0.74	3.18
Winter	1	67	67	0.79	3.31
Exposed					
Summer	1	71	71	0.78	3.31
Winter	2	147	74 (±3)	0.75 (±0.03)	3.24 (±0.21)
Offshore					
Sheltered					
Summer	2	120	60 (±1)	0.66 (±0.02)	2.71 (±0.39)
Winter	2	174	87 (±8)	0.79 (±0.08)	3.51 (±1.36)
Exposed					
Summer	2	147	74 (±3)	0.79 (±0.03)	3.39 (±0.69)
Winter	1	92	92	0.80	3.60
OTUs coral		703			
OTUs water		237			
Shared OTUs		90			
Total no. of OTUs		850			

Overview over sample sites and sampling dates, number of samples, number of operational taxonomic units (OTUs) in coral and water, and evenness and diversity indices.

**Table 4:** Differences in microbial assemblages associated with reef water and the coral *Ctenactis echinata* between study sites and sampling dates

Reef water	d.f.	SS	MS	Pseudo‐F	Unique permutations	Monte Carlo *P*‐value
Shelf	1	1361	1361	1.99	997	0.165
Exposure (shelf)	2	2182	1091	1.59	998	0.230
Season [exposure (shelf)]	4	7183	1796	2.62	999	0.029
Residuals	4	2737	684			
Total	11	13 782				

*C. echinata*	d.f.	SS	MS	Pseudo‐F	Unique permutations	Monte Carlo *P*‐value
Shelf	1	18 485	18485	6.63	997	0.001
Exposure (shelf)	2	16 703	8352	2.99	998	0.001
Season [exposure(shelf)]	4	15 646	3912	1.40	997	0.050
Residuals	34	94 836	2789			
Total	41	150 820				

Results of the permanova analyses showing differences between sampling dates (summer vs. winter) in reef water and coral samples, and between shelf locations (offshore vs. nearshore) and fore‐ and back‐reef environments (i.e. exposed vs. sheltered) in *C. echinata* samples.

### Coral microbiome composition varies over sites and sampling dates

**Figure 4:**
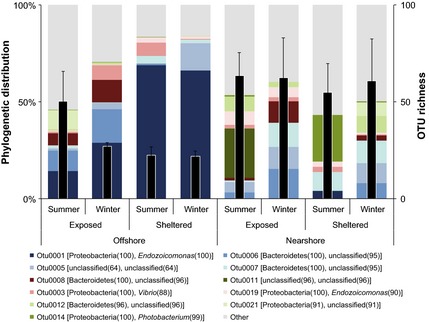
Phylogenetic distribution and operational taxonomic units (OTU) richness of bacteria associated with the coral *Ctenactis echinata* across different habitats. Stacked bars: phylogenetic affiliation of abundant OTUs. Numbers next to phylum and genus denote bootstrap values of classification. Black bars: average number of OTUs associated with *C. echinata*. Error bars: SE.

Of the 703 OTUs associated with coral samples, the 11 most abundant OTUs were encountered on average between 114 and 16 times across all sites and sampling dates. These bacterial taxa accounted for more than 50% of the total bacterial abundance associated with coral samples. Most importantly, the distribution of these abundant taxa differed strongly between sites (Fig. 4). Coral samples from sheltered offshore reef sites were mainly associated with one bacterial taxon (genus *Endozoicomonas*) representing more than 60% of the total microbial assemblage. The same bacterial taxon was also substantially present in coral samples from the exposed counterparts of the offshore reefs, but was almost entirely missing in samples from nearshore reefs (Fig. 4). The remaining 10 bacterial taxa were present at varying degrees over sites and seasons. For instance, a so far uncharacterized bacterium even at the phylum level (OTU0011) was only present at nearshore exposed sites in summer, but with high read numbers (Table S3, Supporting information). In comparison, other OTUs were more evenly distributed across habitats and sampling dates. All but three OTUs remained unclassified at the genus level. Those identified included another taxon of the genus *Endozoicomonas* (OTU0019), one taxon of each the genus *Vibrio* (OTU0003) as well as *Photobacterium* (OTU0014), both of which belong to the family Vibrionaceae (Table S2, Supporting information).

## Discussion

The diversity of coral‐associated microbes is controlled by intrinsic (host‐regulated) as well as external (habitat‐regulated) factors (Thompson *et al*. 2014). Even though a coral host's metabolism contributes to the structure of coral‐associated microbiota (Brown & Bythell 2005), evidence is accumulating that environmental factors such as geographic location, depth, coral and algal cover of the habitat, or elevated temperatures and nutrient concentrations can also influence the coral microbiome (Vega Thurber *et al*. 2009; Kelly *et al*. 2014; Pantos *et al*. 2015). By collecting ecological and molecular data for the coral *Ctenactis echinata* across different habitats and sampling dates (i.e. summer and winter), we were able to derive the distribution range of this coral species and could compare these data to the host‐associated microbial community.

Our data show that bacterial community composition is indicative of a coral's preferred environment (as derived from coral species abundance) and it changes with distance to it. Where *C. echinata* is most abundant, the microbiome is highly structured and dominated by a single bacterial taxon in the genus *Endozoicomonas*. Moving towards habitats where *C. echinata* is less abundant (i.e. potentially marginal habitats), the microbial community assemblage becomes less structured and more diverse. Most importantly, the bacterial community structure aligns with environmental factors including time of the year, water and substrate quality. As such, we argue that the diversity of *C. echinata*'s microbiome correlates with the environmental preference of that coral species and that its level of organization might reflect the distance to the host's preferred environment.

Our study took place during the course of a year representative of Red Sea conditions (Edwards & Head 1987) without bleaching or disease incidents. Therefore, shifts towards disintegrated microbial assemblages dominated by opportunistic or pelagic taxa, as observed during bleaching or disease (Bourne *et al*. 2008; Roder *et al*. 2014), assumingly did not occur and were not visually present. On the molecular level, however, we observed abundance differences of prevailing and rare members of the bacterial community between sampling dates and sites. It is not clear at this point whether these structural differences in the microbial community represent environmental fluctuations or directional adjustments to a more advantageous coral holobiont composition. It is tempting to speculate that changes in the bacterial assembly contribute to phenotypic plasticity by moving the coral holobiont along fitness landscapes (i.e. alternate ‘stable’ states), but further data are needed to unequivocally interrogate such patterns.

More importantly and independent of sampling date influences, patterns of bacterial community structures coincided with the species’ success to prevail at the different sampling locations. *C. echinata* was mostly encountered in habitats with open rocky substrates and clear water conditions (as found along the sheltered sides of offshore reefs). At these sites, the microbiome of *C. echinata* is highly structured (i.e. few OTUs make up the majority of bacterial abundance), with one bacterial taxon of the genus *Endozoicomonas* dominating the bigger part of the bacterial assemblage. With a decrease in rocky substrate availability (as in the exposed sides of the offshore reefs), but similar water quality, this microbial assemblage pattern weakens. Microbial community structure is entirely different in *C. echinata* situated in environments characterized by an increase in loose substrate and/or turbidity in the water column (due to higher TSM concentrations) as prevalent in nearshore reef habitats. A habitat‐discrete association between host and *Endozoicomonas* has also been shown for *Acropora millepora* from the Great Barrier Reef, however, in the opposite direction with nearshore corals holding a stronger structured microbiome and significantly more *Endozoicomonas* compared to midshelf specimens (Lema *et al*. 2014). Following the previous line of argument, *A. millepora*'s preferred habitat might therefore resemble nearshore rather than midshore locations.

Considering that the microbial community is vital for a species’ health (Ezenwa *et al*. 2012), the patterns observed here might hold clues to abundance differences for corals across habitats in the Red Sea and elsewhere. Despite the wide distribution of members of the genus *Endozoicomonas* associated with marine organisms including corals, gorgonians and sponges, among others (Speck & Donachie 2012; Bayer *et al*. 2013a,b; Correa *et al*. 2013; Forget & Kim Juniper 2013; Jessen *et al*. 2013; La Riviere *et al*. 2013; Mendoza *et al*. 2013; Nishijima *et al*. 2013; Pike *et al*. 2013; Rodriguez‐Lanetty *et al*. 2013; Dishaw *et al*. 2014; Ransome *et al*. 2014; Morrow *et al*. 2015), the functional role of this genus is not known. A suggested role is DMSP breakdown (Raina *et al*. 2009, 2010); however, recent comparative sequencing of *Endozoicomonas* genomes isolated from three marine invertebrate hosts confirmed the absence of DMSP‐metabolizing genes in this genus (Neave *et al*. 2014). Other suggested roles include degradation of complex organic carbon sources (La Riviere *et al*. 2013) or the production of antimicrobial compounds (Bourne *et al*. 2008), which has been shown for other coral‐associated bacteria (Ritchie 2006). While their precise function is unknown, current data suggest an important role of *Endozoicomonas* in the coral holobiont.

As with *Endozoicomonas*, elucidation of the role of other abundant bacteria was not possible as for the majority of OTU sequences functional data are absent. Also, 16S rRNA gene similarity to characterized bacteria was on average low prohibiting further functional inference. At present, meta‐analyses using existing data on microbial abundance data in corals and integrating these with collected environmental parameters to interrogate co‐occurrence are most promising, but still rare. We could not retrieve further information for the 10 most abundant OTUs (besides *Endozoicomonas*), other than bacteria from the same genera identified in this study were detected in sea water and coral before.

The increasingly diverse microbial assemblages associated with *C. echinata* sampled outside the species’ preferred habitat indicate a less stable and less structured microbiome, more reminiscent of stressed corals (Bourne *et al*. 2008; Kellogg *et al*. 2013; Roder *et al*. 2014). Interestingly, even for the most abundant OTUs, no taxon was consistently present across all sites and sampling times (Fig. 4). It remains to be determined whether fluctuations in the associated microbiota in different environments are under active host control, environmentally driven or indicative of decreased control of the host over its bacterial symbionts. A recent study by Franzenburg *et al*. (2013) showed that antimicrobial peptides of *Hydra* shape species‐specific bacterial associations, but a similar study in corals is lacking.

In conclusion, we show that microbial communities associated with a coral species comprise a variety of bacterial taxa that differ in abundance and diversity across coral host colonies. Microbial abundance differences align to differences in environmental conditions such as time of year, water quality and substrate availability. In habitats where a coral species is successful (i.e. more abundant), its microbial assemblage appears notably more structured and stable compared to less optimal habitats where key bacterial taxa make way for a less structured community, indicating that ecological niche optimization may shape coral microbiome structure. We can further speculate that the availability of an optimal habitat could be significant for the maintenance of a strongly structured microbiome and its loss might be a key to decreases in coral resilience in habitats of degraded quality or in regard to environmental change.

## Conflict of interest statement

The authors declare that no conflict of interest exists.

C.R. and C.R.V. designed and conceived the experiments. C.R., M.A. and M.K. generated data. C.R., C.R.V. and T.B. analysed the data. C.R. and C.R.V. wrote the manuscript.

## Data accessibility

16S rRNA gene data were uploaded as online Supplementary information. Sequence raw data determined in this study have been deposited in the NCBI Sequence Read Archive under accession no. PRJNA277291.

## Supporting information


**Table S1.** Overview over sampling sites, transect and environmental data and sequence data collection.

**Table S2.** Sequence counts, taxonomic classification and 16S reference amplicon sequence for all OTUs identified and over all samples.

**Table S3.** Differences in substrate cover composition between sampling sites. anosim results comparing substrate cover pairwise and over all sampling sites.
