# Supplementary material for: Microbiome structure of the fungid coral Ctenactis echinata aligns with environmental differences
Source: Mol Ecol. 2015 Jun 19;24(13):3501–11. doi: 10.1111/mec.13251 (PMC4736464; doi:10.1111/mec.13251)
Supplement: Supplementary file 3 — Table S3. Differences in substrate cover composition between sampling sites. anosim results comparing substrate cover pairwise and over all sampling sites. [file MEC-24-3501-s003.docx]

**Supplementary Table 3.** Differences in substrate cover composition between sampling sites. ANOSIM results comparing substrate cover pairwise and over all sampling sites.

| **Pairwise Habitats** | **possible permutations** | **actual permutations** | **R value** | ***p*-value** |
| --- | --- | --- | --- | --- |
| offshore-sheltered vs offshore-exposed | 6435 | 999 | 0.717 | 0.001 |
| offshore-sheltered vs nearshore-sheltered | 495 | 495 | -0.12 | 0.743 |
| offshore-sheltered vs nearshore-exposed | 6435 | 999 | 0.783 | 0.001 |
| offshore-exposed vs nearshore-sheltered | 495 | 495 | 0.752 | 0.002 |
| offshore-exposed vs nearshore-exposed | 6435 | 999 | 0.988 | 0.002 |
| nearshore-sheltered vs nearshore-exposed | 495 | 495 | 0.836 | 0.002 |
| **All Habitats** |  | 999 | 0.726 | 0.001 |
